# Factors related to the outcome of ICU patients with celebral hemorrhage

**DOI:** 10.1186/2197-425X-3-S1-A785

**Published:** 2015-10-01

**Authors:** M Georgiadou, P Trantzas, G Barbarousi, H Pavlou, M Eforakopoulou

**Affiliations:** KAT General Hospital, ICU, Athens, Greece

## Introduction

Intracerebral hemorrhage is a severe condition frequently requiring intensive care admission and mechanical ventilation. Several studies have shown that morbidity and mortality are increased in ICU patients with cerebral hemorrhage.

## Objectives

To investigate the factors associated to the outcome of ICU patients with cerebral hemorrhage.

## Methods

We retrospectively studied 34 patients with cerebral hemorrhage (23 men, 11 women, mean age 64 ± 15 years) hospitalized in ICU of the KAT hospital in Athens. Demographic data of patients, the type, severity and treatment of cerebral hemorrhage, the predisposing factors, the comorbidities, the complications during ICU hospitalization and the outcome were recorded. T-student and chi-square tests were used for statistical analysis.

## Results

Of the studied patients 16 (47%) were improved and left the ICU and 18 (53%) were died. The age, the type of hemorrhage (traumatic, spontaneous, subarachnoid, subdural, intracerebral hematoma) and the type of therapy (surgery or conservative) were not associated with outcome. The Glasgow Coma Scale (GCS) was greater (p < 0.05), whereas the Charlson Comorbidity Index and the SAPS II score were lower in survived patients (p = 0.01 and p = 0.003 respectively). The prior use of anticoagulants as well as prolonged INR and APTT were correlated with mortality (p < 0.05, p = 0.01, p < 0.05 respectively). Also, the recurrence of hemorrhage, liver dysfunction, hyperamylasemia and arrhythmia occurred during ICU hospitalization were associated with poor outcome (p = 0.002, p = 0.01, p < 0.05 respectively).

## Conclusions

ICU patients with cerebral hemorrhage have poor outcome. The coexisting diseases, the previous use of anticoagulants and the complications during ICU hospitalization are associated with mortality.
